# Monoclinic form of (cyanato-κ*N*){2,2′-[ethane-1,2-diylbis(nitrilo­methyl­idyne)]diphenolato-κ^4^ *O*,*N*,*N*′,*O*′}manganese(III)

**DOI:** 10.1107/S1600536810048270

**Published:** 2010-11-24

**Authors:** Daopeng Zhang

**Affiliations:** a College of Chemical Engineering, Shandong University of Technology, Zibo 255049, People’s Republic of China

## Abstract

The title compound, [Mn(C_16_H_14_N_2_O_2_)(NCO)], is a monoclinic polymorph of the previously published ortho­rhom­bic form [Lu *et al.* (2006 ▶). *Inorg. Chem.* **45**, 3538–3548]. The Mn^III^ ion is chelated by a tetra­dentate Schiff base ligand and coordinated by the N atom of a cyanate ligand in a distorted square-pyramidal arrangement. In the crystal, there are short inter­molecular Mn⋯O_phenolate_ distances of 2.752 (3) Å between pairs of inversion-related mol­ecules.

## Related literature

For the ortho­rhom­bic polymorph of the title compound, see: Lu *et al.* (2006 ▶). For related structures, see: Mikuriya *et al.* (1992 ▶); Li *et al.* (1997 ▶); Wang *et al.* (2008 ▶).
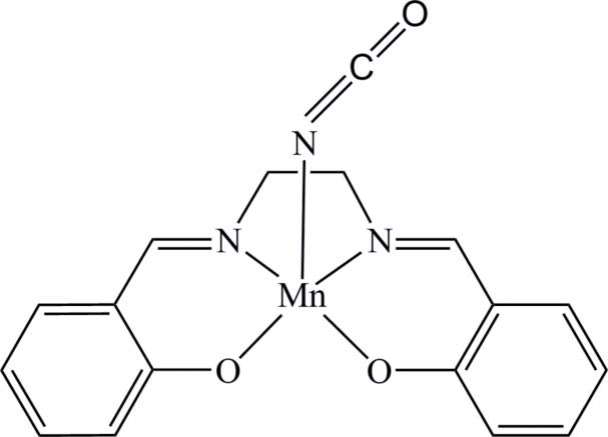


## Experimental

### 

#### Crystal data

[Mn(C_16_H_14_N_2_O_2_)(NCO)]
*M* *_r_* = 363.25
Monoclinic, 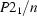

*a* = 9.6399 (16) Å
*b* = 10.9133 (18) Å
*c* = 15.198 (3) Å
β = 97.826 (3)°
*V* = 1584.0 (5) Å^3^
*Z* = 4
Mo *K*α radiation
μ = 0.85 mm^−1^
*T* = 293 K
0.37 × 0.35 × 0.23 mm

#### Data collection

Bruker APEXII CCD area-detector diffractometer
Absorption correction: multi-scan (*SADABS*; Sheldrick, 1996 ▶) *T*_min_ = 0.743, *T*_max_ = 0.828
7377 measured reflections
2698 independent reflections
1769 reflections with *I* > 2σ(*I*)
*R*_int_ = 0.047

#### Refinement

*R*[*F* ^2^ > 2σ(*F* ^2^)] = 0.048
*wR*(*F* ^2^) = 0.131
*S* = 1.01
2698 reflections
217 parameters
H-atom parameters constrained
Δρ_max_ = 0.53 e Å^−3^
Δρ_min_ = −0.33 e Å^−3^

### 

Data collection: *APEX2* (Bruker, 2004 ▶); cell refinement: *SAINT-Plus* (Bruker, 2001 ▶); data reduction: *SAINT-Plus*; program(s) used to solve structure: *SHELXS97* (Sheldrick, 2008 ▶); program(s) used to refine structure: *SHELXL97* (Sheldrick, 2008 ▶); molecular graphics: *SHELXTL* (Sheldrick, 2008 ▶); software used to prepare material for publication: *SHELXTL*.

## Supplementary Material

Crystal structure: contains datablocks I, global. DOI: 10.1107/S1600536810048270/lh5164sup1.cif

Structure factors: contains datablocks I. DOI: 10.1107/S1600536810048270/lh5164Isup2.hkl

Additional supplementary materials:  crystallographic information; 3D view; checkCIF report

## Figures and Tables

**Fig. 1.**
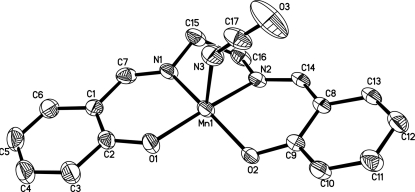
The molecular structure of the title complex with the atom-labelling scheme. Displacement ellipsoids are drawn at the 30% probability level. H atoms are not shown.

## supplementary crystallographic information

### Comment

Because of their excellent chelating ability for metal atoms, the tetradentate schiff-base ligands containing N_2_O_2_ coordination unit have been widely studied in coordination chemistry field. Here, we report a Mn(III) complex based on tetradentate ligand *N*,*N*'-bis(salicylidene)-1,2-diphenyl-1,2-ethanediamine.

The molecular structure of the title complex is shown in Figure 1. The Mn^III^ ion is involved in a distorted square-pyramidal arrangement by a N_3_O_2_ unit, in which the four basal sites are occupied by two N atoms and two O atoms from the Schiff base ligand, and the apical position is occupied by the N atom of a cyanato ligand. The bond distances can be compared to this those found in the related structures (Lu, *et al.*, 2006; Mikuriya, *et al.*, 1992; Li, *et al.*, 1997; Wang, *et al.*, 2008). The Mn^III^ ion lies above the basal plane formed by N_2_O_2_ unit by 0.228 Å. The short intermolecular distance of Mn···O_phenolate_ 2.752 (3) Å indicates that there exsits weak interaction between the two complexes realted by inversion centers in the crystal.

### Experimental

The synthesis of the title complex was carried out by mixing Mn(ClO_4_)_2_.6H_2_O (0.1mmol), NaNCO (0.1mmol) and the schiff-base ligand (0.1mmol) in methanol (20ml). The mixture was stirred for about half an hour at room temperature and then filtered and the filtrate allowed to partially evaporate in air for sevral days to produce crystals suitable for X-ray diffraction with a yield about 64%.

### Refinement

All the H atoms bonded to the C atoms were placed using the HFIX commands in *SHELXL-97* (Sheldrick, 2008) with C—H distances of 0.93 and 0.97 Å, respectively, and were allowed for as riding atoms with *U*_iso_(H) = 1.2*U*_eq_(C).

### Crystal data

**Table:**

[Mn(C_16_H_14_N_2_O_2_)(NCO)]	*F*(000) = 744
*M**_r_* = 363.25	*D*_x_ = 1.523 Mg m^−^^3^
Monoclinic, *P*2_1_/*n*	Mo *K*α radiation, λ = 0.71073 Å
Hall symbol: -P 2yn	Cell parameters from 1289 reflections
*a* = 9.6399 (16) Å	θ = 2.6–26.6°
*b* = 10.9133 (18) Å	µ = 0.85 mm^−^^1^
*c* = 15.198 (3) Å	*T* = 293 K
β = 97.826 (3)°	Block, dark-brown
*V* = 1584.0 (5) Å^3^	0.37 × 0.35 × 0.23 mm
*Z* = 4	

### Data collection

**Table:**

Bruker APEXII CCD area-detector diffractometer	2698 independent reflections
Radiation source: fine-focus sealed tube	1769 reflections with *I* > 2σ(*I*)
graphite	*R*_int_ = 0.047
φ and ω scans	θ_max_ = 24.7°, θ_min_ = 2.3°
Absorption correction: multi-scan (*SADABS*; Sheldrick, 1996)	*h* = −11→11
*T*_min_ = 0.743, *T*_max_ = 0.828	*k* = −8→12
7377 measured reflections	*l* = −17→17

### Refinement

**Table:**

Refinement on *F*^2^	Primary atom site location: structure-invariant direct methods
Least-squares matrix: full	Secondary atom site location: difference Fourier map
*R*[*F*^2^ > 2σ(*F*^2^)] = 0.048	Hydrogen site location: inferred from neighbouring sites
*wR*(*F*^2^) = 0.131	H-atom parameters constrained
*S* = 1.01	*w* = 1/[σ^2^(*F*_o_^2^) + (0.0695*P*)^2^] where *P* = (*F*_o_^2^ + 2*F*_c_^2^)/3
2698 reflections	(Δ/σ)_max_ = 0.001
217 parameters	Δρ_max_ = 0.53 e Å^−^^3^
0 restraints	Δρ_min_ = −0.33 e Å^−^^3^

### Special details

**Table:**

Geometry. All e.s.d.'s (except the e.s.d. in the dihedral angle between two l.s. planes) are estimated using the full covariance matrix. The cell e.s.d.'s are taken into account individually in the estimation of e.s.d.'s in distances, angles and torsion angles; correlations between e.s.d.'s in cell parameters are only used when they are defined by crystal symmetry. An approximate (isotropic) treatment of cell e.s.d.'s is used for estimating e.s.d.'s involving l.s. planes.
Refinement. Refinement of *F*^2^ against ALL reflections. The weighted *R*-factor *wR* and goodness of fit *S* are based on *F*^2^, conventional *R*-factors *R* are based on *F*, with *F* set to zero for negative *F*^2^. The threshold expression of *F*^2^ > σ(*F*^2^) is used only for calculating *R*-factors(gt) *etc*. and is not relevant to the choice of reflections for refinement. *R*-factors based on *F*^2^ are statistically about twice as large as those based on *F*, and *R*- factors based on ALL data will be even larger.

### Fractional atomic coordinates and isotropic or equivalent isotropic displacement parameters (Å^2^)

**Table:**

	*x*	*y*	*z*	*U*_iso_*/*U*_eq_	
Mn1	0.10463 (6)	0.63253 (5)	0.52050 (4)	0.0420 (2)	
O1	−0.0313 (3)	0.7121 (2)	0.57698 (17)	0.0489 (7)	
O2	0.1015 (3)	0.4886 (2)	0.58923 (17)	0.0472 (7)	
O3	0.5003 (4)	0.6657 (5)	0.6799 (3)	0.1254 (17)	
N1	0.0793 (3)	0.7500 (3)	0.4211 (2)	0.0453 (8)	
N2	0.2150 (3)	0.5430 (3)	0.4394 (2)	0.0425 (8)	
N3	0.2893 (4)	0.7093 (3)	0.5904 (2)	0.0566 (10)	
C1	−0.1070 (4)	0.8699 (4)	0.4711 (3)	0.0476 (10)	
C2	−0.1116 (4)	0.8071 (4)	0.5515 (3)	0.0463 (10)	
C3	−0.2089 (5)	0.8453 (4)	0.6060 (3)	0.0621 (13)	
H3	−0.2123	0.8054	0.6598	0.075*	
C4	−0.2991 (5)	0.9395 (4)	0.5824 (4)	0.0715 (14)	
H4	−0.3638	0.9619	0.6196	0.086*	
C5	−0.2956 (5)	1.0021 (4)	0.5037 (4)	0.0724 (15)	
H5	−0.3568	1.0667	0.4879	0.087*	
C6	−0.2003 (4)	0.9674 (4)	0.4492 (3)	0.0605 (12)	
H6	−0.1974	1.0096	0.3963	0.073*	
C7	−0.0106 (4)	0.8380 (4)	0.4105 (3)	0.0530 (11)	
H7	−0.0132	0.8849	0.3593	0.064*	
C8	0.3143 (4)	0.3997 (3)	0.5504 (3)	0.0415 (9)	
C9	0.2170 (4)	0.4207 (4)	0.6109 (3)	0.0423 (9)	
C10	0.2394 (4)	0.3634 (4)	0.6929 (3)	0.0503 (10)	
H10	0.1770	0.3776	0.7334	0.060*	
C11	0.3501 (4)	0.2868 (4)	0.7163 (3)	0.0626 (13)	
H11	0.3630	0.2503	0.7721	0.075*	
C12	0.4445 (4)	0.2632 (4)	0.6557 (3)	0.0638 (13)	
H12	0.5195	0.2103	0.6709	0.077*	
C13	0.4252 (4)	0.3189 (4)	0.5740 (3)	0.0524 (11)	
H13	0.4872	0.3025	0.5337	0.063*	
C14	0.2980 (4)	0.4541 (4)	0.4634 (3)	0.0451 (10)	
H14	0.3511	0.4225	0.4220	0.054*	
C15	0.1771 (4)	0.7294 (4)	0.3564 (3)	0.0569 (12)	
H15A	0.2662	0.7686	0.3763	0.068*	
H15B	0.1395	0.7634	0.2992	0.068*	
C16	0.1964 (4)	0.5929 (4)	0.3489 (3)	0.0533 (11)	
H16A	0.1150	0.5565	0.3141	0.064*	
H16B	0.2780	0.5752	0.3201	0.064*	
C17	0.3891 (6)	0.6904 (5)	0.6329 (3)	0.0659 (14)	

### Atomic displacement parameters (Å^2^)

**Table:**

	*U*^11^	*U*^22^	*U*^33^	*U*^12^	*U*^13^	*U*^23^
Mn1	0.0320 (4)	0.0509 (4)	0.0445 (4)	0.0037 (3)	0.0098 (3)	0.0052 (3)
O1	0.0417 (16)	0.0560 (18)	0.0502 (17)	0.0124 (14)	0.0108 (13)	0.0024 (13)
O2	0.0324 (16)	0.0555 (17)	0.0570 (19)	0.0080 (13)	0.0174 (13)	0.0130 (13)
O3	0.051 (2)	0.238 (5)	0.081 (3)	0.023 (3)	−0.012 (2)	−0.046 (3)
N1	0.0299 (18)	0.057 (2)	0.049 (2)	0.0001 (16)	0.0062 (15)	0.0068 (16)
N2	0.0301 (18)	0.057 (2)	0.042 (2)	0.0001 (16)	0.0086 (14)	0.0047 (16)
N3	0.043 (2)	0.065 (3)	0.059 (3)	−0.0037 (19)	0.0009 (19)	−0.0029 (19)
C1	0.034 (2)	0.045 (2)	0.063 (3)	−0.003 (2)	0.0041 (19)	0.001 (2)
C2	0.035 (2)	0.046 (2)	0.057 (3)	−0.001 (2)	0.004 (2)	−0.010 (2)
C3	0.056 (3)	0.063 (3)	0.069 (3)	0.008 (2)	0.015 (2)	−0.007 (2)
C4	0.053 (3)	0.068 (3)	0.095 (4)	0.014 (3)	0.014 (3)	−0.007 (3)
C5	0.052 (3)	0.054 (3)	0.109 (5)	0.015 (2)	0.003 (3)	−0.006 (3)
C6	0.044 (3)	0.056 (3)	0.080 (3)	0.002 (2)	0.001 (2)	0.011 (2)
C7	0.038 (2)	0.060 (3)	0.059 (3)	−0.007 (2)	−0.001 (2)	0.016 (2)
C8	0.028 (2)	0.050 (2)	0.048 (2)	−0.0009 (17)	0.0096 (17)	−0.0012 (19)
C9	0.030 (2)	0.048 (2)	0.049 (3)	−0.0021 (18)	0.0077 (18)	0.0004 (19)
C10	0.034 (2)	0.068 (3)	0.049 (3)	0.000 (2)	0.0102 (18)	0.005 (2)
C11	0.040 (3)	0.087 (4)	0.059 (3)	0.008 (2)	0.004 (2)	0.023 (3)
C12	0.034 (3)	0.080 (3)	0.078 (3)	0.011 (2)	0.008 (2)	0.016 (3)
C13	0.028 (2)	0.065 (3)	0.066 (3)	0.000 (2)	0.013 (2)	0.003 (2)
C14	0.026 (2)	0.059 (3)	0.052 (3)	−0.0040 (19)	0.0122 (18)	−0.006 (2)
C15	0.040 (3)	0.081 (3)	0.051 (3)	0.008 (2)	0.013 (2)	0.022 (2)
C16	0.039 (2)	0.085 (3)	0.037 (2)	0.003 (2)	0.0105 (18)	0.005 (2)
C17	0.048 (3)	0.100 (4)	0.054 (3)	−0.013 (3)	0.021 (3)	−0.029 (3)

### Geometric parameters (Å, °)

**Table:**

Mn1—O1	1.874 (2)	C5—C6	1.371 (6)
Mn1—O2	1.889 (3)	C5—H5	0.9300
Mn1—N1	1.971 (3)	C6—H6	0.9300
Mn1—N2	1.991 (3)	C7—H7	0.9300
Mn1—N3	2.118 (4)	C8—C13	1.394 (5)
O1—C2	1.320 (4)	C8—C9	1.418 (5)
O2—C9	1.340 (4)	C8—C14	1.438 (5)
O3—C17	1.235 (6)	C9—C10	1.386 (5)
N1—C7	1.288 (5)	C10—C11	1.364 (5)
N1—C15	1.469 (5)	C10—H10	0.9300
N2—C14	1.280 (5)	C11—C12	1.403 (6)
N2—C16	1.466 (5)	C11—H11	0.9300
N3—C17	1.102 (6)	C12—C13	1.372 (5)
C1—C6	1.403 (5)	C12—H12	0.9300
C1—C2	1.408 (5)	C13—H13	0.9300
C1—C7	1.437 (6)	C14—H14	0.9300
C2—C3	1.397 (5)	C15—C16	1.508 (6)
C3—C4	1.363 (6)	C15—H15A	0.9700
C3—H3	0.9300	C15—H15B	0.9700
C4—C5	1.382 (7)	C16—H16A	0.9700
C4—H4	0.9300	C16—H16B	0.9700
			
O1—Mn1—O2	93.85 (11)	N1—C7—C1	125.7 (4)
O1—Mn1—N1	91.64 (12)	N1—C7—H7	117.1
O2—Mn1—N1	162.75 (13)	C1—C7—H7	117.1
O1—Mn1—N2	167.59 (13)	C13—C8—C9	119.1 (4)
O2—Mn1—N2	89.12 (12)	C13—C8—C14	118.8 (3)
N1—Mn1—N2	82.24 (13)	C9—C8—C14	122.0 (3)
O1—Mn1—N3	100.33 (13)	O2—C9—C10	119.5 (3)
O2—Mn1—N3	97.30 (13)	O2—C9—C8	122.1 (3)
N1—Mn1—N3	97.78 (14)	C10—C9—C8	118.3 (4)
N2—Mn1—N3	91.22 (14)	C11—C10—C9	122.1 (4)
C2—O1—Mn1	130.4 (2)	C11—C10—H10	119.0
C9—O2—Mn1	121.3 (2)	C9—C10—H10	119.0
C7—N1—C15	120.9 (3)	C10—C11—C12	119.8 (4)
C7—N1—Mn1	126.2 (3)	C10—C11—H11	120.1
C15—N1—Mn1	112.9 (2)	C12—C11—H11	120.1
C14—N2—C16	122.7 (3)	C13—C12—C11	119.5 (4)
C14—N2—Mn1	124.2 (3)	C13—C12—H12	120.3
C16—N2—Mn1	113.0 (2)	C11—C12—H12	120.3
C17—N3—Mn1	145.7 (4)	C12—C13—C8	121.2 (4)
C6—C1—C2	118.7 (4)	C12—C13—H13	119.4
C6—C1—C7	118.8 (4)	C8—C13—H13	119.4
C2—C1—C7	122.5 (4)	N2—C14—C8	124.2 (3)
O1—C2—C3	118.3 (4)	N2—C14—H14	117.9
O1—C2—C1	123.4 (3)	C8—C14—H14	117.9
C3—C2—C1	118.2 (4)	N1—C15—C16	107.4 (3)
C4—C3—C2	121.6 (5)	N1—C15—H15A	110.2
C4—C3—H3	119.2	C16—C15—H15A	110.2
C2—C3—H3	119.2	N1—C15—H15B	110.2
C3—C4—C5	120.8 (5)	C16—C15—H15B	110.2
C3—C4—H4	119.6	H15A—C15—H15B	108.5
C5—C4—H4	119.6	N2—C16—C15	107.2 (3)
C6—C5—C4	118.9 (4)	N2—C16—H16A	110.3
C6—C5—H5	120.5	C15—C16—H16A	110.3
C4—C5—H5	120.5	N2—C16—H16B	110.3
C5—C6—C1	121.8 (5)	C15—C16—H16B	110.3
C5—C6—H6	119.1	H16A—C16—H16B	108.5
C1—C6—H6	119.1	N3—C17—O3	178.2 (6)
